# Improvement of arterial oxygenation in free-ranging moose *(Alces alces)* immobilized with etorphine-acepromazine-xylazine

**DOI:** 10.1186/s13028-014-0051-5

**Published:** 2014-08-15

**Authors:** Marianne Lian, Alina L Evans, Mads F Bertelsen, Åsa Fahlman, Henning A Haga, Göran Ericsson, Jon M Arnemo

**Affiliations:** 1 Center for Zoo and Wild Animal Health, Copenhagen Zoo, Frederiksberg, DK-2000, Denmark; 2 Department of Forestry and Wildlife Management, Faculty of Applied Ecology and Agricultural Sciences, Hedmark University College, Campus Evenstad, Koppang, NO-2480, Norway; 3 Section of Arctic Veterinary Medicine, Department of Food Safety and Infection Biology, Norwegian School of Veterinary Science, Tromsø, NO-9292, Norway; 4 Department of Clinical Sciences, Faculty of Veterinary Medicine and Animal Science, Swedish University of Agricultural Sciences, Uppsala, SE-750 07, Sweden; 5 Section of Anesthesia and Radiology, Department of Companion Animal Clinical Sciences, Norwegian School of Veterinary Science, Oslo, NO-0033, Norway; 6 Department of Wildlife, Fish and Environmental Studies, Faculty of Forest Sciences, Swedish University of Agricultural Sciences, Umeå, SE-901 83, Sweden

**Keywords:** Acid–base status, Alces alces, Arterial blood gases, Atipamezole, Etorphine, Hypoxemia, Immobilization, Moose, Xylazine

## Abstract

**Background:**

The effect of intranasal oxygen and/or early reversal of xylazine with atipamezole on arterial oxygenation in free-ranging moose (*Alces alces*) immobilized with etorphine-acepromazine-xylazine with a cross-sectional clinical study on 33 adult moose was evaluated.

Moose were darted from a helicopter with 3.37 mg etorphine, 15 mg acepromazine and 75 mg xylazine. Intranasal oxygen at a flow rate of 4 L/min and/or early reversal of xylazine with 7.5 mg atipamezole to improve oxygenation was evaluated, using four treatment regimens; intranasal oxygen (n = 10), atipamezole intramuscularly (n = 6), atipamezole intravenously (n = 10), or a combination of atipamezole intravenously and intranasal oxygen (n = 7). Arterial blood was collected 7–30 minutes (min) after darting, and again 15 min after institution of treatment and immediately analyzed using an i-STAT®1 Portable Clinical Analyzer.

**Results:**

Before treatment the mean ± SD (range) partial pressure of arterial oxygen (P_aO2_) was 62 ± 17 (26–99) mmHg. Twenty-six animals had a P_aO2_ < 80 mmHg. Ten had a P_aO2_ of 40–60 mmHg and three animals had a P_aO2_ < 40 mmHg. Intranasal oxygen and intravenous administration of atipamezole significantly increased the mean P_aO2_, as did the combination of the two. In contrast, atipamezole administered intramuscularly at the evaluated dose had no significant effect on arterial oxygenation.

**Conclusions:**

This study shows that intranasal oxygen effectively improved arterial oxygenation in immobilized moose, and that early intravenous reversal of the sedative component, in this case xylazine, in an opioid-based immobilization drug-protocol significantly improves arterial oxygenation.

## Background

Research to improve moose management, health and welfare often requires chemical immobilization. In Scandinavia approximately 5,000 immobilizations of moose have been carried out since 1984 for ecological studies and management purposes. Although capture mortality rates for moose in Norway and Sweden have been low, from 0.5% - 1.0% [1], the non-lethal adverse effects of capture and immobilization procedures have traditionally been ignored. A recent study from Sweden showed that female moose changed movement patterns for 4.5 days post immobilization with opioid combinations [2]. Another study demonstrated that moose cows immobilized during the last three months of pregnancy gave birth to calves with reduced postnatal survivorship [3].

Potent opioids or opioids in combination with sedatives are most commonly used to immobilize moose. Etorphine, carfentanil and thiafentanil have all been used successfully [4]–[7]. When opioids are used alone, moose are conscious and responsive to stimulation, but usually immobilized sufficiently for approach and handling. These drugs are, however, major respiratory depressants [8],[9]. Hypoxemia is a known problem in wildlife immobilization [10]. Inadequate oxygen delivery will lead to tissue hypoxia causing cell damage to vital tissues like brain, myocardium, kidneys and liver [11],[12]. Markedly decreased arterial oxygen partial pressure (P_aO2_) levels indicating severe hypoxemia has been documented in several wildlife species during immobilization. This seems to be most evident in wildlife species immobilized with opioids, with reported P_aO2_ values as low as 10 mmHg in rhebok (*Pelea capreolus*) [8] and <40 mmHg in species such as North American elk (*Cervus canadensis*), white rhinoceros (*Ceratotherium simum*), white-tailed deer (*Odocoileus virginianus*), impala (*Aepyceros melampus*), black rhinoceros (*Diceros bicornis*) and wood bison (*Bison bison athabascae*) [13]–[19].

Moose immobilized with etorphine, xylazine and acepromazine demonstrated severe hypoxemia, with the lowest P_aO2_ at 36 mmHg, and marked acidemia (pH < 7.20) [9], emphasizing the need for research into prevention of hypoxemia in immobilized moose. Nasal oxygen insufflation has been shown to alleviate hypoxemia associated with chemical immobilization in several wild ungulate species [14]–[17],[20], but to our knowledge, has not previously been evaluated in free-ranging moose. A combination of etorphine, acepromazine and xylazine has been used for immobilization of moose in Sweden since 1979 [1],[21]. Opioids and alpha-2 adrenoceptor agonists are respiratory depressant drugs [12]. Further, xylazine is a vasoconstrictor, resulting in bradycardia, reduced cardiac output and a biphasic blood pressure response. The biphasic blood pressure response leads to a transient hypertension followed by a prolonged hypotension [22]. Complete reversal of the etorphine would end the immobilization, whereas reversal of the xylazine was hypothesized to improve respiration while maintaining immobilization. Evans *et al.*[9] conducted a physiological evaluation of free-ranging moose immobilized with this drug protocol in Sweden. They found severe hypoxemia in all animals immediately after recumbency, and found no improvement in P_aO2_ values in the second sample collected after 15 minutes of down time. In this study we evaluated intranasal oxygen insufflation and partial reversal of the immobilization protocol using atipamezole, as measures against hypoxemia in moose immobilized with etorphine, acepromazine and xylazine. Based on the study done in 2012, we did not find it ethical to include a control group receiving no treatment [9].

## Methods

The study was conducted during February 2012 in Öster Malma, Södermans län (58.95° N, 17.16° E) and Växjö, Kronobergs län (56.87° N, 14.80° E), Sweden. Both locations have altitudes less than 170 m above sea level.

Thirty female and three male moose, 1–18 years old captured for collaring and biometrics were sampled. Initially only females were included. However, both genders were included in the last treatment group (the combination of intranasal oxygen and atipamezole intravenously), to increase the sample size for this group. Only animals sufficiently immobilized (sternal recumbency, staying down when approached) with one dart were included in the study. Animals were aged based on tooth wear [23]. Ambient temperature and barometric pressure (P_B_) were recorded. All captures had ethical approval from the Ethical Committee on Animal Experiments in Umeå, Sweden (Umeå Djurförsöksetiska Nämnd), ethical permit number DNR A 50–12.

The drug mixture was made by adding 10 mL Large Animal Immobilon® (Novartis Animal Health, Litlington, UK, 2.25 mg/mL etorphine and 10 mg/mL acepromazine) to one vial of Rompun® dry powder (xylazine, Bayer AG, Leverkusen, Germany, 500 mg). Darts were loaded with1.5 mL of the mixture, resulting in doses of 3.37 mg etorphine, 15 mg acepromazine and 75 mg xylazine. Moose were located using a helicopter and darted with 3-mL Dan inject darts with 2.0 x 40-mm barbed needles with side-ports, in gluteal or epaxial muscles with a CO_2_ powered rifle (Dan-Inject, Børkop, Denmark) from an estimated distance of three to ten meters. Needle size was selected based on season and expected body condition to ensure a complete intramuscular (i.m.) injection. All darts had a Recco® tracking device (Recco AB, Lidingö, Sweden) and all darts were recovered. Xylazine was reversed with atipamezole (Antisedan®, 5 mg/mL Orion Pharma Animal Health, Turku, Finland) at a dose ratio of 0.1 mg per mg xylazine intravenously (i.v.) or i.m. Atipamezole was administered either for early reversal of xylazine at 13–31 min (mean 21 min) after darting or i.v. at 40–60 min after darting when etorphine was reversed with diprenorphine (Large Animal Revivon® 3 mg/ml, Novartis Animal Health, Litlington, UK) at a dose ratio of 1.34 mg per mg etorphine giving a total dose of 4.5 mg. The animals receiving atipamezole i.m. were not given additional atipamezole i.v. at the time of opioid reversal.

Variables recorded included time from sighting to successful darting (chase time), time from darting to recumbency (induction time), time from recumbency to reaching and handling the animal (capture time), time from darting until reversal agent was administered (reversal time) and time from administration of diprenorphine to standing (recovery time). These variables are summarised in Table 1. Induction and recovery quality were assessed subjectively. Moose found in lateral recumbency at capture, were placed in sternal recumbency. For those with the head on the ground, snow was cleared from around the nostrils. Pulse rate was measured by palpation of the auricular artery, respiration rate by counting thoracic elevations and rectal temperature with a digital thermometer. Capillary refill time (CRT) and mucus membrane color were monitored from the mucus membrane of the eye. Jaw tone was used to assess muscle tone and classified as absent, tense or rigid. Palpebral reflex (absent, present or spontaneous) and presence of movement were also monitored. All variables were measured when the animal was first approached after recumbency, and repeated 15 min later. Rectal temperature was not repeated in female moose, since they were rectally palpated to establish reproductive status.

**Table 1:** Time variables recorded and compared for each treatment

***Variable***	***Units***	**Oxygen treatment**	**Atipamezole i.m.**	**Atipamezole i.v.**	**Combination treatment**
Chase time	minutes	3.6 ± 3.2 (1–12)	3.8 ± 2.3 (1–8)	4.6 ± 3.0 (1–9)	3.4 ± 1.8 (2–7)
Induction time	minutes	7.1 ± 3.6 (2–13)	7.2 ± 4.6 (2–15)	7.8 ± 4.3 (4–15)	9.1 ± 4.6 (3–16)
Capture time	minutes	6.5 ± 3.3 (3–13)	6.2 ± 1.7 (4–8)	5.8 ± 1.6 (3–9)	5.3 ± 1.9 (3–8)
Reversal time	minutes	34.4 ± 3.4 (31–41)	46.5 ± 9.2 (40–53)	34.3 ± 2.0 (32–38)	34.7 ± 3.9 (30–41)
Recovery time	seconds	122 ± 24 (93–160)	131 ± 27 (100–170)	109 ± 17 (72–125)	109 ± 18 (90–135)

Time from sighting to successful darting (chase time), time from darting to recumbency (induction time), time from recumbency to reaching and handling the animal (capture time), time from darting until reversal agent was administered (reversal time) and time from administration of diprenorphine to standing (recovery time) in free-ranging moose *(Alces alces)* darted from helicopter with etorphine-acepromazine-xylazine. Mean ± SD (range) values are presented.

Degree of central nervous system depression was classified as level I (mildly affected, voluntary movement and intact reflexes), level II (no voluntary movement and intact reflexes), level III (unconsciousness, depressed reflexes, muscular relaxation) and level IV (ceased respiration, dilated pupils). This evaluation was assessed at capture, and again 15 minutes after treatment.

As soon as possible after capture and 15 min after start of treatment an arterial blood sample was collected anaerobically from the auricular artery, using a pre-heparinized syringe (Portex® Arterial blood gas sampling kit, Smiths Medical ASD, New Hampshire, USA) and a 23-gauge needle. The sample was analyzed immediately using an i-STAT®1 Portable Clinical Analyzer and i-STAT® CG4+ and 6+ cartridges (Abbott Laboratories, Illinois, USA). The analyzer was kept in an insulated box with warm water bottles to maintain the temperature between 16 - 30°C.

Measured variables included pH, P_aO2_, partial pressure of carbon dioxide (P_aCO2_) and lactate. P_aO2_, P_aCO2_ and pH were corrected based on rectal temperature. The initial temperature was used for correction of both samples in all animals. Calculated values included bicarbonate (HCO_3_^−^), hemoglobin oxygen saturation (SaO_2_) and base excess (BE).

The alveolar-arterial oxygen tension difference (P_(A-a)O2_) prior to oxygen insufflation was estimated for the temperature corrected values, based on calculation of the alveolar oxygen tension (PAO_2_) calculated from the alveolar gas equation [PAO_2_ = FiO_2_ (PB - P_H2O_ ) - (PaCO_2_/RQ)], where F_iO2_ = fraction of inspired oxygen (0.21) and P_H2O_ = saturated vapor pressure for water at 37°C (47 mmHg). The respiratory quotient (RQ) was assumed to be 1 for moose [24]. P_(A-a)O2_ was not calculated after oxygen insufflation, because the FiO2 was then unknown.

For the first part of the study moose were randomly assigned to one of three different treatments, intranasal oxygen insufflation from a portable oxygen cylinder at a flow rate of 4 L/min (oxygen group), early administration of atipamezole i.m. with a 20 gauge needle and 3 ml syringe in femoral muscles (i.m. group) or an early administration of atipamezole i.v. with a 20 gauge needle and 3 ml syringe in the jugular vein (i.v. group). Having completed 10 animals with intranasal oxygen treatment, six animals with atipamezole i.m. and 10 animals with atipamezole i.v., it was decided to include a fourth group treated with a combination of intranasal oxygen at a flow rate of 4 L/min and atipamezole intravenously (combination group).

The oxygen nasal line was inserted 10 cm into one of the nostrils and fixated with a clothes peg or tape. Fifteen min after starting treatment, a second arterial blood sample was collected and analysed in the same way. Venous blood samples for serum biochemistry were collected from the jugular vein before treatment was initiated.

Statistics were done using JMP® (SAS Campus Drive, North Carolina, USA). The Shapiro-Wilk test for normality was used to confirm that the data (recovery time, P_aO2_, P_aCO2_, pH, lactate, pulse and respiratory rates) within each treatment group was normally distributed. Within each group, the difference between the first and second sample was analyzed using a two-tailed paired t-test. A simple Bonferroni adjustment was used (0.05/7), and therefore *P* values less than 0.007 were considered significant. To compare the mean increase in P_aO2_ from the first to the second sample from the four groups, oneway ANOVA (Analysis of Variance) was used. Comparison for all pairs was done using Tukey-Kramer HSD. Simple descriptive statistics including mean, standard deviation and range for all physiological variables was calculated in Microsoft® Excel® 2007. Mean ± SD (range) values are presented.

## Results

Ambient temperature was −6.7 ± 5 (−19.0 – 2.3)°C. The barometric pressure ranged from 723 to 781 mmHg.

The induction time was 7.5 ± 4 (2–16) minutes. At capture, 21 moose showed no movement, 11 moved their head and one moved its head and neck. Palpebral reflex was absent in 14 moose, present in 16 moose and three moose were blinking spontaneously. Muscle tone was absent in 17 moose, while 16 moose had muscle tone intact. Generalized tremors were observed in two animals with muscle tone. All moose were sufficiently immobilized for handling with a moderate degree of central nervous system depression (stage I to III) and their heads were lowered. All animals were alive four months after capture. Chase time, capture time, induction time, reversal time and recovery time for each group are presented in Table 1. Each group is presented with physiological variables and blood gasses before and after 15 minutes of treatment in Table 2.

**Table 2:** Physiological variables from immobilized moose

			**Oxygen**	**Oxygen**		**Atipamezole i.m.**	**Atipamezole i.m.**		**Atipamezole i.v.**	**Atipamezole i.v.**		**Combination**	**Combination**
			**Before**	**After 15 min of O2**		**Before**	**15 min after**		**Before**	**15 min after**		**Before**	**15 min after**
Variable	Unit	N	T1: 7–27 minutes	T2: 22–45 minutes	N	T1: 11–29 minutes	T2: 27–44 minutes	N	T1: 11–29 minutes	T2: 26–45 minutes	N	T1: 13–30 minutes	T2: 28–45 minutes
Rectal temp	C	10	38.2 ± 0.7 (37.5 - 39.4)	Not recorded	6	37.8 ± 0.3 (37.3 - 38.0)	Not recorded	8	38.1 ± 0.8 (37.7 - 39.3)	Not recorded	7	38.1 ± 0.2 (37.9 - 38.4)	Not recorded
Pulse rate	Beats/min	10	50 ± 9 (40–66)	38 ± 7 (24–48)^‡^	6	44 ± 6 (36–52)	37 ± 4 (30–40)^‡^	8	47 ± 9 (36–60)	46 ± 7 (40–56)	7	50 ± 15 (36–64)	48 ± 14 (22–64)
Resp rate	Breaths/min	10	34 ± 9 (12–45)	29 ± 8 (18–44)	6	29 ± 3 (24–32)	25 ± 4 (20–28)	8	32 ± 6 (20–40)	24 ± 6 (16–36)^‡^	7	30 ± 6 (20–36)	23 ± 8 (12–36)
Capillary refill time	seconds	8	1.3 ± 0.5 (1–2)	1 ± 0 (1)	6	1.5 ± 0.5 (1–2)	1 ± 0 (1)	8	1.9 ± 0.3 (1–2)	1 ± 0 (1)	7	1.6 ± 0.5 (1–2)	1 ± 0 (1)
Color mucus membrane	1-4^†^	8	3 (1–4)	3 (2–4)	6	2 (1–3)	3 (2–4)	8	3 (2–4)	4 (3–4)	7	3 (2–4)	4 (3–4)
P_aO2_*	mmHg	10	67 ± 19 (32–99)	127 ± 38 (63–185)^‡^	6	61 ± 19 (40–86)	67 ± 23 (41–109)	8	55 ± 18 (26–84)	67 ± 14 (46–86)^‡^	7	61 ± 13 (47–80)	96 ± 20 (75–124)^‡^
P_aO2_*	Kpa	10	8.9 ± 2.5 (4.2 - 13.2)	16.9 ± 5.0 (8.4 - 24.7)^‡^	6	8.1 ± 2.5 (5.3 - 11.5)	8.9 ± 3.1 (5.5 - 14.5)	8	7.3 ± 2.4 (3.5 - 11.2)	8.9 ± 1.9 (6.1 - 11.5)^‡^	7	8.1 ± 1.8 (6.3 - 10.7)	12.8 ± (10.0 - 16.6)^‡^
P_aCO2_*	mmHg	10	56.6 ± 9.3 (47.1 - 76.4)	68.6 ± 13.3 (51.6 - 91.9)^‡^	6	63.4 ± 8.7 (50.0 - 71.8)	67.3 ± 6.4 (58.7 - 75.5)	8	60.0 ± 9 (48.2 - 73.8)	62.2 ± 8.1 (52.1 - 77.1)	7	57.6 ± 7.7 (48.5 - 67.4)	68.2 ± 8.1 (54.6 - 76.8)^‡^
P_aCO2_*	kPa	10	7.5 ± 1.2 (6.3 - 10.2)	9.1 ± 1.8 (6.9 - 12.1)^‡^	6	8.5 ± 1.2 (6.7 - 9.6)	9.0 ± 0.9 ( 7.8 - 10.1)	8	8.0 ± 1.2 (6.4 - 9.8)	8.3 ± 1.1 (6.9 - 10.3)	7	7.8 ± 1.0 (6.5 - 9.0)	9.1 ± 1.1 (7.3 - 10.2)^‡^
pH*		10	7.28 ± 0.07 (7.15 - 7.37)	7.27 ± 0.09 (7.17 - 7.42)	6	7.26 ± 0.05 (7.20 - 7.33)	7.29 ± 0.03 (7.25 - 7.34)	8	7.29 ± 0.05(7.23 - 7.36)	7.33 ± 0.04 (7.28 - 7.39)	7	7.23 ± 0.13(7.01 - 7.37)	7.24 ± 0.1 (7.06 - 7.38)
Lactate	mmol/L	10	7.1 ± 4.3 (2.4 - 16.6)	4.9 ± 3.6 (1.6 - 13.5)^‡^	6	5.3 ± 1.3 (3.9 - 7.4)	3.3 ± 0.5 (2.7 - 3.9)^‡^	8	5.0 ± 2.7 (3.3 - 11.5)	2.5 ± 0.8 (1.5 - 4.0)^‡^	7	9.1 ± 6.2 (0.6 - 18.2)	5.6 ± 4.6 (0.6 - 14.4)^‡^

Physiological variables from free-ranging moose *(Alces alces),* during chemical immobilization with etorphine-acepromazine-xylazine, delivered by dart syringe from helicopter. Mean ± SD (range) values are presented for all variables, except color of mucus membrane, where median (range) is presented. ^†^: 1: blue, 2: blue-pink, 3: pale pink, 4: pink. *: temperature corrected values. ^‡^: significant difference after treatment. T1 is time from darting to collection of first sample. T2 is time from first to second sample.

The initial P_aO2_ for all moose was 62 ± 17 (26–99) mmHg. Twenty-six out of 33 moose had a P_aO2_ < 80 mmHg (26–77 mmHg) before treatments were initiated. Ten animals had a P_aO2_ between 40 – 60 mmHg and three animals had a P_aO2_ < 40 mmHg. The P_(A-a)O2_ was 30 ± 12 (7–58) mmHg before treatments (sample 1).

Lactate was 6.8 ± 4.1 (0.6 - 18.2) mmol/L in the first sample, and all four groups showed a significant decline in lactate from the first to the second sample. P_aCO2_ was > 45 mmHg in all animals, and in 14 animals P_aCO2_ was between 60 – 80 mmHg. Twenty-nine animals had a pH < 7.35 before treatment, and of these, five had a pH < 7.20.

In the i.m. group two out of six animals had an increase in P_aO2_, with 14 and 58 mmHg after treatment. In three animals P_aO2_ decreased. No increase in movement was observed, and the degree of central nervous system depression was assessed to be the same through the immobilization period. All animals in the i.m. group had poor recoveries, with trouble standing.

Two outliers were removed from the i.v. group, one which fell into lateral recumbency during the immobilization period and one in which intravenous injection was not complete and partly administrated subcutaneously. P_aO2_ increased significantly (*P* = 0.004) after treatment, with a mean increase of 12 mmHg. However, six animals still had low P_aO2_-values (P_aO2_ < 80 mmHg). At the time of the second sample palpebral reflex or spontaneous blinking were present in all animals. They regained muscle tone, moved their head, and were able to hold the head elevated from the ground. All animals experienced smooth recoveries. This was also seen in the combination group, where all animals also regained palpebral reflex or were blinking spontaneously, regained muscle tone, moved their head and held it elevated from the ground, and had smooth recoveries.

There was no significant difference in recovery time among groups, but a trend toward faster recovery for moose in the two groups treated with an early intravenous reversal of xylazine. The mean increase in P_aO2_ between samples was significantly (*P* = 0.0008) different between the intranasal oxygen group and the two atipamezole groups, while the mean increase in P_aO2_ did not differ significantly between the intranasal oxygen group and the combination group. Mean increase of P_aCO2_ was not significantly different in any of the groups.

## Discussion

This study documented the effect of intranasal oxygen insufflation and early reversal of xylazine with atipamezole on improving arterial oxygenation in chemically immobilized free-ranging moose.

The mild to severe hypoxemia seen in 26 out of 33 moose was expected based on a previous report of hypoxemia in moose immobilized with this combination [9]. At sea level in normally ventilating mammals breathing air, P_aO2_ is between 80 – 100 mmHg [12]. In our study, seven out of 33 moose had an assumed normal P_aO2_ in the first arterial blood sample. There was evidence of impaired oxygen exchange before onset of treatment, with a P_(A-a)O2_ of 30 ± 12 (7–58) mmHg. However, with values ranging from 7 – 58 mmHg, this was not evident in all moose. The concurrent high respiratory rate might have been a sign of superficial breathing, and thereby increasing dead space ventilation and lowering the amount of alveolar oxygen. Since the study was conducted at low altitudes, reduced inspired pressure of oxygen can be ruled out as primary cause of hypoxemia. Recognized side effects of opioid and alpha-2 adrenoceptor agonists in ruminants are hypoventilation and intrapulmonary factors like increased diffusion barrier, lower V/Q ratio and increased physiological shunt [12],[25],[26]. Since all individuals were hypercapnic in the first sample with an increased mean P_(A-a)O2_, hypoventilation and intrapulmonary factors were likely major contributors to the hypoxemia in the immobilized moose in this study.

Hypoxemia in large animals may also be a result of recumbency. In conscious horses [27] and cattle [28] it has been shown that P_aO2_ decreases when the animals position is changed from sternal to lateral or dorsal position. Even in sternal recumbency the abdominal viscera may compress the diaphragm, leading to low V/Q mismatch which result in venous admixture [12].

In the two treatment groups given oxygen, P_aO2_, increased in all individuals and there was a significant increase in the mean P_aO2_ between the first and the second sample. P_aCO2_ also increased significantly after oxygen treatment. This has been reported previously in wild cervids receiving oxygen supplementation [14],[16],[17] Ventilation is mainly controlled by central chemoreceptors in the medulla sensitive to pH and P_aCO2_ and peripheral chemoreceptors in the aortic and carotid bodies sensitive to pH and P_aO2_. However, the response of these chemoreceptors to P_aO2_, P_aCO2_ and pH is depressed by opioids and sedatives. Further, the increased P_aO2_ levels arising from the oxygen treatment diminishes the respiratory drive caused by hypoxemia, resulting in an increased level of hypoventilation and P_aCO2_[12]. This probably resulted in the significant increase in P_aCO2_ seen in the two groups given oxygen. The Haldane effect may also have contributed to the significant increase in P_aCO2_ in these groups, since increasing oxygenation of blood decreases the capacity for carbon dioxide transport. When the arterial oxygenation improves in the hypoxemic patient, the affinity of carbon dioxide for hemoglobin decreases and carbon dioxide is easily displaced. This causes the overall P_aCO2_ in the blood to increase as the arterial oxygenation improves [14],[16],[17],[29]. Studies on elk [17] and bongo antelopes (*Tragelaphus eurycerus*) [20] found the same effect with a concurrent decreased respiratory rate. In the current study, there was a significantly decreased respiratory rate in the i.v. group. Interestingly, in the two atipamezole groups that did not receive intranasal oxygen, the increase in P_aCO2_ between samples was not significant. However, the four groups were not significantly different when compared statistically. To reduce the high P_aCO2_ levels found with this drug protocol, endotracheal intubation and intermittent positive-pressure ventilation would be necessary [30].

When atipamezole was administered intramuscularly only one animal showed a satisfactory increase in arterial oxygenation, from 51 mmHg to 109 mmHg. Furthermore, there was no change in movement, and only a slight improvement in mucus membrane color and capillary refilling time was observed in the animals. This was likely a result of the degree of central nervous system depression remaining the same, and, therefore few changes in the alpha-2 adrenoceptor agonist effects on circulation. The lack of effect in this treatment group was probably a result of insufficient time and effectiveness for atipamezole to be absorbed from the muscle. This is supported by poor recoveries with difficulty standing and avoiding trees in this group, likely due to residual effects of xylazine. Based on these findings we decided to discontinue the i.m. group after a sample size of six animals.

There was a significant increase in P_aO2_ between the first and second sample when atipamezole was administered intravenously. By reversing the xylazine component of the immobilization protocol, moose went from level II – III of central nervous depression to level I. A more superficial level of immobilization eliminated some of the drug induced depression on circulation and breathing reflexes. After antagonizing xylazine, the moose were able to hold their head elevated off the ground. Improved head holding behavior ensured clear airways and probably facilitated gas exchange. Antagonizing the cardiovascular side effects from xylazine improved mucus membrane color and capillary refilling time, reflecting better circulation. Better circulation may have improved pulmonary perfusion by increasing the V/Q ratio, leading to the significantly increased P_aO2_. However, only two out of eight animals improved their P_aO2_ values high enough to be considered as normal values.

In a field situation, with limited personnel and equipment, chemical methods for improving respiration would be advantageous. In this study, early reversal of xylazine appears to improve gas exchange. Alpha-2 adrenoceptor agonists can have serious side effects including bloat, regurgitation and aspiration, leading some authors to not recommend the combination of xylazine with opioids for immobilization of wild ruminants [6], however early reversals of these drugs would allow for their beneficial effects during induction, while avoiding potential side effects during the immobilization period.

Arterial oxygenation was not significantly different between the oxygen group and the combination group. Although, in the combination group moose regained palpebral reflex and muscle tone and were able to hold their head off the ground, similar to moose in the i.v. group. The two groups with early intravenous reversal of xylazine had significantly improved P_aO2_ values despite no change in P_aCO2_, and improved head holding behavior, which suggest better gas exchange with a decrease in degree of central nervous system depression. Capillary refilling time normalized in both groups, and color of mucus membranes improved in both groups. By reversing xylazine, the vascular side effects were probably decreased, leading to an improved circulation [22].

Acidemia was documented in most animals, likely caused both by hypercapnia and anaerobic metabolism. An increase in blood CO_2_ decreases pH, which decreases hemoglobin affinity for oxygen (Bohr Effect). The resulting release of oxygen is beneficial for hypoxic tissues. However, this negatively influences pulmonary hemoglobin oxygen uptake. And, a severe acidemia decreases myocardial contractility and predisposes to arrhythmias [31]. The pH decreased over time in the oxygen group. In the two groups with an early intravenous reversal of xylazine pH had a trend of increasing over time, however, this was not significant. Acidemia has been reported from other studies on oxygen supplement to free-ranging ungulates [8],[17],[19].

Lactate is an anaerobic metabolic product, and the plasma concentration increases when tissue is deprived of oxygen [31]. In a study of moose immobilized with etorphine, elevated lactate levels were positively correlated with physical exertion (snow depth) and short induction time (dose dependent) [32]. Elevated lactate levels have been suspected to be one of the factors leading to capture myopathy [33]. Two independent studies, found hyperlactemic moose immobilized with both etorphine alone [32] and the combination of etorphine, acepromazine and xylazine [9]. Mortality due to capture myopathy has not been reported with the etorphine protocol used in Norway. However, with the etorphine, acepromazine and xylazine protocol used in Sweden, there have been at least two deaths related to capture myopathy [1] . All animals in this study significantly decreased lactate levels between the first and the second sample. The elevated lactate level was probably related to lactic acid accumulation due to muscle activity during darting and induction. A decreased delivery of oxygen to tissues due to low P_aO2_ would also contribute to the high lactate levels found in this study. However, the decrease in lactate concentration over time seen in this study indicates that the cellular environment in the muscle tissue improved [31].

## Conclusions

This study showed that moose immobilized with etorphine, acepromazine and xylazine have low arterial oxygenation, and varying degree of respiratory and metabolic acidemia. Nasal insufflation with oxygen proved to be a simple and effective technique to improve arterial oxygenation.

Further, this study showed that when the immobilizing drugs for moose consist of a potent opioid combined with xylazine, it is beneficial for the arterial oxygenation to reverse xylazine immediately after the animal becomes recumbent. Atipamezole should be administered intravenously, to have a significant effect on PaO_2_.

The recommended treatment for hypoxemia in moose immobilized with etorphine, acepromazine and xylazine is the combination of intranasal oxygen and an early intravenous reversal of the alpha-2 adrenoceptor agonist.

## Competing interests

The authors declare that they have no competing interests.

## Authors’ contributions

ML carried out fieldwork and data collection, drafted the manuscript and did statistical analysis. ALE carried out fieldwork, did statistical analysis and revised the manuscript. MFB planned study design, revised the manuscript and helped with interpretation of results. ÅF carried out fieldwork, revised the manuscript and helped with interpretation of results. HAH revised the manuscript and helped with interpretation of results. GE facilitated and planned fieldwork and revised the manuscript. JMA facilitated and planned fieldwork, planned study design and revised the manuscript. All authors have critically revised the manuscript and read and approved the final manuscript.
